# Phytoremediation of Potentially Toxic Elements: Role, Status and Concerns

**DOI:** 10.3390/plants12030429

**Published:** 2023-01-17

**Authors:** Zishan Ahmad Wani, Zeeshan Ahmad, Mohd Asgher, Jahangeer A. Bhat, Manju Sharma, Ashish Kumar, Virbala Sharma, Amit Kumar, Shreekar Pant, Alexander S. Lukatkin, Naser A. Anjum

**Affiliations:** 1 Conservation Ecology Lab, Department of Botany, Baba Ghulam Shah Badshah University, Rajouri 185234, JK, India; 2 Department of Plant Sciences, Quaid-i-Azam University, Islamabad 45320, Pakistan; 3 Plant Physiology and Biochemistry Laboratory, Department of Botany, School of Biosciences and Biotechnology, Baba Ghulam Shah Badshah University, Rajouri 185234, JK, India; 4 College of Horticulture & Forestry, Rani Lakshmi Bai Central Agricultural University, Jhansi 284003, UP, India; 5 Department of Environmental Science, Baba Ghulam Shah Badshah University, Rajouri 185234, JK, India; 6 G. B. Pant National Institute of Himalayan Environment, Garhwal Regional Centre, Srinagar Garhwal 246174, UK, India; 7 Department of Environmental Sciences, Central University of Himachal Pradesh, Dharamsala 176213, HP, India; 8 School of Hydrology and Water Resources, Nanjing University of Information Science and Technology, Nanjing 210044, China; 9 Centre for Biodiversity Studies, Baba Ghulam Shah Badshah University, Rajouri 185234, JK, India; 10 Department of General Biology and Ecology, N.P. Ogarev Mordovia State University, Bolshevistskaja Str., 68, Saransk 430005, Russia; 11 Plant Physiology and Biochemistry Laboratory, Department of Botany, Aligarh Muslim University, Aligarh 202002, UP, India

**Keywords:** potentially toxic elements, pollution, hazardous effects, phytoremediation, plant biomass

## Abstract

Environmental contamination with a myriad of potentially toxic elements (PTEs) is triggered by various natural and anthropogenic activities. However, the industrial revolution has increased the intensity of these hazardous elements and their concentration in the environment, which, in turn, could provoke potential ecological risks. Additionally, most PTEs pose a considerable nuisance to human beings and affect soil, aquatic organisms, and even nematodes and microbes. This comprehensive review aims to: (i) introduce potentially toxic elements; (ii) overview the major sources of PTEs in the major environmental compartments; (iii) briefly highlight the major impacts of PTEs on humans, plants, aquatic life, and the health of soil; (iv) appraise the major methods for tackling PTE-caused pollution; (v) discuss the concept and applications of the major eco-technological/green approaches (comprising phytoextraction, rhizofiltration, phytostabilization, phytovolatilization, and phytorestoration); (vi) highlight the role of microbes in phytoremediation under PTE stress; and (vii) enlighten the major role of genetic engineering in advancing the phytoremediation of varied PTEs. Overall, appropriate strategies must be developed in order to stop gene flow into wild species, and biosafety issues must be properly addressed. Additionally, consistent efforts should be undertaken to tackle the major issues (e.g., risk estimation, understanding, acceptance and feasibility) in order to guarantee the successful implementation of phytoremediation programs, raise awareness of this green technology among laymen, and to strengthen networking among scientists, stakeholders, industrialists, governments and non-government organizations.

## 1. Introduction

Anything that gets accumulated in the natural environment beyond its permissible limit causes deleterious effects on the biotic as well as abiotic components of the ecosystem [1]. The metal contaminant known as heavy metals is one of these examples. Metalloids, transition metals, actinides, and lanthanides are all part of the subset of elements known as heavy metals that have metallic properties. The term heavy metal is generally used for metallic elements with specific weights over 5 g/cm^3^ and are noxious even at low concentrations [2]. To date, 23 metals have been classified as heavy metals, with the most common being lead (Pb), cadmium (Cd), cobalt (Co), chromium (Cr), and mercury (Hg), which have potentially toxic effects at high and low concentrations [3]. These toxic elements are classified as class-B and come under non-essential elements [4,5]. These metals persist in the surroundings and accumulate at different levels of the food chain, from primary producer level to consumer levels, through consumption due to their non-biodegradable nature (unlike that of organic pollutants); thus, these are deleterious for both components (biotic as well as abiotic) of an ecosystem [6,7]. Hence, it is important for environmentalists to take strategic remediation measures to reduce heavy metals’ load in the aquatic and terrestrial environments [8]. Pourret and Hursthouse [9] prefer the term ‘potentially toxic elements (PTEs)’ for such elements instead of ‘heavy metals’ due to their toxic nature. The accumulation of toxic metals in the ecosystem is continually being fueled by the agricultural use of fertilizers and pesticides, as well as industrial inputs and metal-contaminated sewage [10]. This makes it difficult to use soils effectively and safely. Furthermore, environmental heavy metal pollution posed a grave threat to human life as well as the other biotic elements of the ecosystem. Hence, it is important for environmentalists to take strategic remediation measures to reduce heavy metals’ load in aquatic and terrestrial environments [11].

To date, numerous remediation approaches have been implemented to treat heavy metal contamination in aquatic and terrestrial soil but phytoremediation is reported to be the most efficient technique among these (e.g., physiochemical, mechanical, etc.). Phytoremediation is defined as the utilization of plants for in situ management of contaminated soils, water and sediments. The methods for reducing load contaminants via phytoremediation entail phytoextraction, phytostabilization, rhizofiltration, and phytovolatilization [12]. There are certain advantages to using phytoremediation [7]: (i) economically feasible (i.e., simple to manage, and installation and maintenance cost is very low); (ii) eco-friendly; (iii) wider applicability; (iv) it prevents metals from leaching;(v) risk of spreading metal contaminants is low; and (vi) improves soil fertility and makes the soil healthy for plant growth. The literature reveals that various studies have been conducted in the recent past to improve the efficiency of phytoremediation but it is still a frequently discussed topic in the scientific community.

This review paper aimed to assess the sources, impact and different approaches for tackling hazardous levels of PTE pollution. Different approaches of phytoremediation are discussed and an inventory of plant species with phytoremediation potential is presented. The review also highlights the future prospects of phytoremediation, with a wider application of genetic engineering. Thus, this paper provides baseline information for the environmentalist to improve the performance of plants for the phytoremediation process.

## 2. Sources of Heavy Metals or PTEs

**Figure 1:**
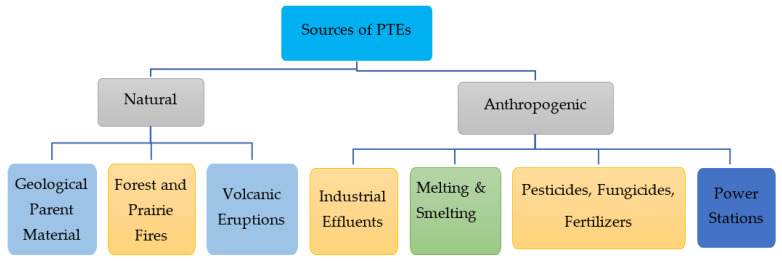
Different natural and anthropogenic sources of heavy metals or PTEs in the environment.

PTEs, or heavy metals, come from both natural and anthropogenic sources (Figure 1). Among natural sources, geologic parent rock material is the most considerable one. PTEs released from geologic parent rocks are determined through rock type and the environmental conditions that trigger the weathering process. A significant amount of manganese (Mn), copper (Cu), nickel (Ni) and chromium (Cr) is contributed by various igneous rocks, such as augite, hornblende and olivine, etc. The copious concentrations of aluminum (Al), Mn, Pb, Ni, Cu, and Hg and other noxious gases are emanated into the environment from volcanic activities coupled with air-borne releases of PTEs originating from the forest fire [13]. Significant concentrations of PTEs originate from anthropogenic resources, comprising industrial waste, refining procedures, mining, furnaces, martial tasks, and synthetic compounds in agricultural processes [14]. Mining, refinement, smelting, transportation of ores and other industrial activities add ample amounts of PTEs to the environment and these activities have tainted broad areas throughout the world. Power plants using petroleum and coal combustion, and nuclear power stations also add PTEs to the environment. Further, organic and inorganic fertilizers coupled with other agricultural activities, such as liming, sewage outflow, sludge, irrigation water, pesticides, and fungicides, also cause the PTE contamination of an ecosystem [14]. With the increasing use of pesticides and fertilizers, coupled with the discharge from smelting industries and metalliferous mines, PTEs have tainted large areas of land [15]. Annually, thousands of metric tons of PTEs are added to the environment from different refineries and industrial units worldwide [16].

## 3. Major Impacts of PTEs

During the last few decades, the risk of living organisms to PTE toxicity has increased due to the increase in anthropogenic activities, industrialization, and modern agricultural practices [17,18]. Due to the persistent and stable nature of PTEs, they cannot be degraded or destroyed [19]. PTEs are bio-accumulative and may gradually invade biological systems through air, water, and the series of the food chain over a definite period [20]. PTE contagion is a major ecological concern that influences the wellbeing of plants, animals, and humans, and affects the quality of the environment.

### 3.1. Impacts on Human

Some heavy metals have immense bio-significance to human, such asiron (Fe), zinc (Zn) and Mn. Their daily therapeutic and nutritional allowances had been suggested for good human health. However, some of these, such as arsenic (As), Pb, Cd and Hg in methylated form, have deleterious influence even at a very small amount [21]. Sources and effects of some of the PTEs are given in Table 1. PTEs such as Cd, Pb and Hg demonstrate an extensive toxicity level against biological entities, including human beings [22]. Inhalation and ingestion are the pathways through which these metals invade the human body. Hence, these lead to the diminution of some significant nutrients in the body, which consequently diminishes immunological defenses, cause growth retardation, and may increase gastrointestinal and other cancer rates [23,24,25]. Exposure to Cd and Pb might cause impaired fertility in humans. Cd is considered a metal estrogen. It can link and stimulate the alpha and beta estrogen receptors and concurrently cause the stimulation of progesterone receptors; thus, it may be designated a probable contributory mediator of estrogen-linked ailments, such as endometrial and breast cancer, endometriosis, and spontaneous abortion [26]. Pb can directly lead to an elevated risk of impulsive abortion through its possible teratogenic action [27]. Further, PTEs are probably the agents affecting the neurological system, renal functioning, and ossification process. These also attach to phosphate, deoxyribose sugar, and heterocyclic base residues of DNA, resulting in mutations by inducing alterations in DNA structures [24]. Long-term exposure to PTEs results in severe neurological diseases such as Alzheimer’s, Parkinson’s, muscular dystrophy and sclerosis.

### 3.2. Impacts on Plants

**Figure 2:**
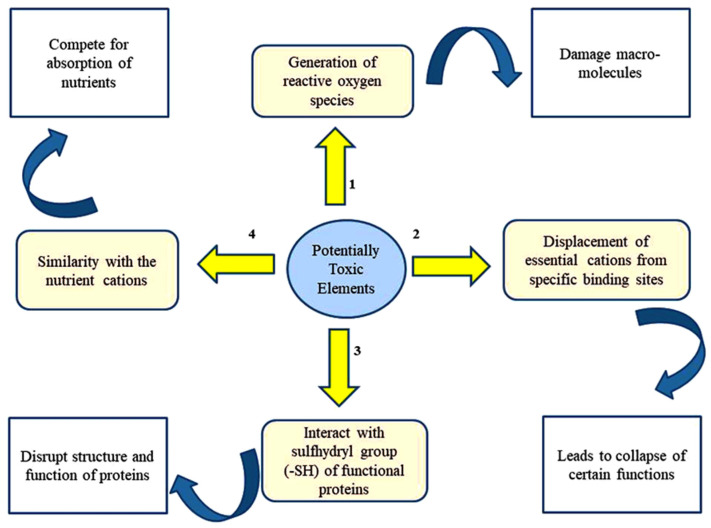
Major mechanisms underlying PTE-caused toxicity in plants.

**Figure 3:**
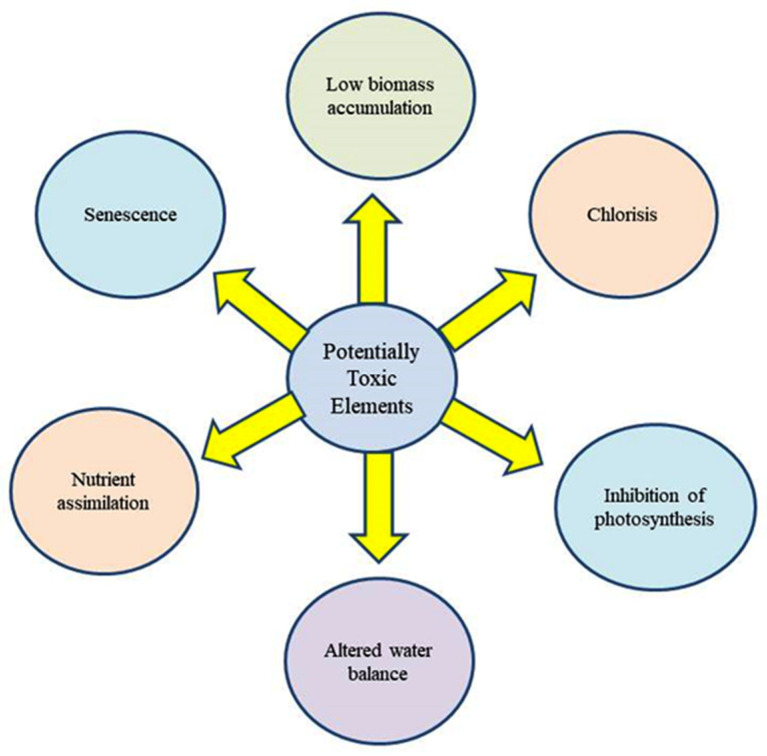
Major toxic effects of PTEs on plants.

There are four proposed mechanisms through which PTEs exert toxicities in plants [35,36] (Figure 2). In addition, PTEs affect many physiological and developmental processes in plant biosystems (Figure 3).

The main entry points for PTE ions into plant systems are the leaves and roots. High PTE concentrations have detrimental effects, including the inhibition of cytoplasmic enzymes and oxidative stress-related cell structure damage [37]. Additionally, PTEs have detrimental effects on the development and activity of soil microorganisms, which ultimately may affect plant growth. Further, due to PTEs’ interference with the activities of soil microbes, enzyme activities important for plant metabolisms may also be hindered. PTEs are also attributed with increasing enzyme activity, such as glucose-6-phosphate dehydrogenase, and peroxides in the leaves of plants growing in contaminated soils [38]. Further, PTEs are also responsible for stomatal closure, increased ethylene production and cause iron deficiency by inhibiting uptake from the medium or due to immobilization in root tissues [39]. PTEs such as Cd, As, Cr, and Cu inhibit PSII and photosynthetic genes (*psbA* and *psbB*), and increased accumulation of PTEs in roots, stems and leaves causes reduced growth in plants [40,41,42]. Aluminum damages root tips by stimulating the synthesis and accumulation of callose by inhibiting the assembly and release of border cells [43].Further, Al can accrue in plant cells and have the potential to bind at multiple sites, such as cell wall, cell membrane and nucleic acids, and impede various biological, physiological and cellular processes [43]. Pb is one of the most prevalent toxic elements in the soil, is widely dispersed and harms the various enzymatic activities in plants [44]. PTE toxicity causes a reduction in the decomposition of litter, a reduction in the mineralization of carbon and nitrogen, and an inhibition of microbial growth [19]. Plants growing in PTE-contaminated soil experience both reduced growth and yield, as the PTEs hinder growth and developmental processes [45]. PTE toxicity has a variety of effects on plants at the cellular and molecular levels. PTEs, for example, affect germination, chlorophyll biosynthesis, photosynthesis, exchange of gases, respiration, blocking functional groups of crucial molecules for metabolism, and processes of the central dogma of life [46]. These toxic effects cause overall plant growth to slow down, which may eventually cause the plant to die [44].

### 3.3. Impact on Aquatic Life

Due to PTEs’ toxic nature, accumulation/biomagnification, and level of environmental persistence, the accumulation of PTEs in fresh and marine water systems is one of the principal hazards for aquatic organisms [47]. The main sources of these toxic metals in aquatic ecosystems are biological activities such as weathering, soil leaching, urban storms, as well as anthropogenic activities such as land filling, coal mining, agricultural activities, and effluent discharges [48]. PTEs can take on many different forms in aquatic systems, including being adsorbed on particulate matter, precipitating as insoluble salts, or existing in an ionic state. However, all forms are harmful to aquatic life, especially the benthic fauna, and may cause several oxidative stresses in those living things [49]. Whole plant or animal bodies are exposed to PTE toxicity in aquatic ecosystems. PTEs in the water are especially harmful to young fish and may even lead to the extinction of the entire fish population in contaminated reservoirs [50].

### 3.4. Impacts on Soil Health

Different levels of heavy metal concentration are measured as one of potent soil contaminants and their elevated concentrations may decrease soil productivity to a great extent by harming soil flora and fauna, which, in turn, causes alterations in the size of the population and community composition of soil microbes, and by altering the physical and chemical status of soil, including increasing soil acidity, redox potential, and enhanced decomposition of organic matter. Through other means as well, contaminants have the potential to cause shifts in microbial populations, according to the isolation-based technique [49]. Generally, an increase in PTE concentration in soil negatively affects soil microbial estates and causes an overall decrease in the growth of microbes by affecting their growth, morphology and metabolism [51,52]. PTEs are also toxic for nematodes and the most toxic PTE for nematodes is selenium (Se) [53].

## 4. Methods to Tackle PTE Pollution

### 4.1. Conventional Methods

#### 4.1.1. Chemical Precipitation

Chemical precipitation (CP) includes the expansion of synthetic reagents and coagulants, such as lime, alum, iron salts and natural polymers, followed by accelerated solids from cleaned water. CP takes place at basic pH within the pH range of 9–11 [54]. The advantage of using CP is its uncomplicated, cheap, and non-toxic procedure. However, it requires a sufficient amount of chemical reagents to diminish cation-charged metals to a tolerable limit of eviction. It is ineffective at low concentrations of contaminants and requires an oxidative step when contaminants are complex; it involves both a high sludge production, and handling and disposal problems [55].

#### 4.1.2. Electro Dialysis

Electro dialysis (ED) is a layer procedure through which particles are moved across specifical film (cation and anion particular), affected by an electric potential [56]. Cation-particular films are the polyelectrolytes with the contrarily charged matter, which dismisses negatively charged particles and lets unambiguously charged particles move through and the other way around. Limitations of this method are: (i) economic constraints; (ii) rapid saturation of ion exchangers; and (iii) its time-consuming nature [55].

#### 4.1.3. Coagulation/Flocculation

Coagulation is a chemical response that ensues as a coagulant (e.g., alum, polyaluminum chloride, ferric sulfate, ferric chloride, cationic polydiallyldimethylammonium chloride, polyacrylamide) supplemented with water [57]. It supports colloidal constituents in water to consolidate into small “flocs”. The suspended material is then heaved into these flocs. The process of encouraging flocs to form and grow to a size that will easily settle is called flocculation. This water-blending process is moderately complex. As a pretreatment for other processes, such as advanced oxidation, membrane filtration, adsorption, or ion exchange, coagulation/flocculation procedure is used frequently [57]. In these methods, non-reusable chemicals must be added, effluents must be monitored through physiochemical parameters, and sludge volume must be increased [55].

#### 4.1.4. Ultrafiltration

Ultrafiltration (UF) is a detachment procedure utilizing layers with pore sizes ranging from 0.1 to 0.001 micron [56]. Usually, UF evacuates high-level atomic weight substances, colloidal and natural plus synthetic polymeric particles. It is the weight propelled size prohibition sanitization procedure in which low atomic weight elements and water saturate a film, while macromolecule, colloids and particles are held. UF can remove metals with an efficiency of more than 90% when the metal concentration is between 10 and 112 mg/L, the pH is between 5 and 9.5, and the pressure is between 2 and 5 bars, depending on the membrane characteristics [56]. This method is expensive with high energy requirements, rapid membrane clogging and limited flow rates [55].

#### 4.1.5. Reverse Osmosis (RO)

In reverse osmosis (RO), uncontaminated water and spoiled water are separated by layers resembling cellophane. In this procedure, pressure is applied to the film’s concentrated side while purified water is forced into the film’s weakened side, washing away the concentrated side’s pollutants [58]. RO can be used to remove biological, suspended, and soluble contaminants from water. It involves a diffusive mechanism; thus, the solute concentration, pressure, and water flux rate all play a role in severance competence [56]. Limitations of this method are: (i) huge energy requirements, and (ii) its expensive nature [55].

#### 4.1.6. Adsorption

Adsorption is a procedure that takes place when a gaseous or fluid solute is hoarded on the outside of an adsorbent, framing a nuclear/subatomic film. It is employable in physical, organic and substance frameworks. It is usually utilized in charcoal, pitches’ manufacturing and water decontamination [59]. Due to its efficiency and affordability, adsorption is a popular wastewater treatment method [60]. It is a commonly used method for removing metal particles from various effluent types [61], and chemically altered plant products such as activated carbon are primarily used as an adsorbent that can remove a variety of dangerous metals [62]. In addition to activated/modified plant products, a number of other substances, such as natural zeolites, activated carbons, sepiolite, and kaolin, can also function as adsorbents [62]. Limitations of this method are: (i) expensive nature; (ii) requirements of several types of adsorbents; (iii) non-selective method; and (iv) rapid saturation of reactors [55].

## 5. Eco-Technological/Green Approach

Due to their sessile nature, plants are unable to escape sudden or unfavorable environmental changes. PTE toxicity causes several physiological and biochemical changes, and plants must develop defensive mechanisms to deal with these adverse effects [63]. In response to the increased levels of PTEs in the media, plants develop homeostatic contrivances to retain appropriate concentrations of metallic ions within cell organelles in order to curtail the mutilations caused by the toxic levels of PTEs. Thus, the absorption, allocation, and detoxification of such metallic ions are under the control of a controlled network of transport and chelation [64,65]. There are several mechanisms through which plants react to PTEs toxicity, viz., detecting external stressors, transmitting a signal into the cell through signal transduction, and enacting the necessary actions to mitigate the adverse effects of stressors by modifying the physiology, biochemistry, as well as molecular status of the cell [17]. Some higher plant species, hyperaccumulators, can accrue these toxic elements within their tissues and are resistant to PTE toxicity [66].

Additionally, by exuding enzymes and root exudates, some plants can promote the breakdown of PTEs in the rhizosphere. Such plant species can be used effectively to eradicate PTEs from the environment because they have the capacity to absorb, accumulate, degrade, and thus lower the hazardous level of PTEs from the medium. Using plants to treat polluted soils and water is a multidisciplinary approach known as phytoremediation, which works best when the pollutants are widespread and located in the plant’s root zone [67,68]. Phytoremediation-based strategies are treated as green technologies that are cost effective and include phytoextraction, phytovolatilization, phytostabilization, and rhizofiltration [69]. Numerous types of contaminants, pollutants, or hazardous materials have been subjected to the phytoremediation method in both laboratory-based studies and the natural environment [70,71,72]. Although the phytoremediation was distinguished and recognized by humans some 300 years ago, its efficient approach and extension were not evaluated until the 1980s [29]. With the advent of more evolved technological processes, the biological and engineering approaches were conceived to advance the utility of phytoremediation to minimize the concentration of hazardous materials in filthy media. Nowadays, it is an eco-technological approach in which natural and genetically modified plant species are used to accrue, degrade, or trim down hazardous materials to their abilities [73].

A member of the Brassicaceae family, the genus *Brassica*, produces a large volume of biomass and is reliable in a variety of environmental conditions, making it an effective remediation candidate [74,75].Further, high concentration of thiocyanates in many other species of the family Brassicaceae, known to be hyperaccumulators of PTEs, make them unpalatable to animals, thus reducing the accessibility of PTEs at different trophic levels [76]. On the other hand, trees and agricultural crops tend to retain comparatively lower amounts of PTE than hyperaccumulators despite their high biomass [77]. Most of the angiosperms are terrestrial, though some are aquatic; thus, angiosperms are suitable for the remediation of both soil and aqueous media. On the other hand, pteridophytes prefer aquatic or moist habitats and are, thus, more suitable for the remediation of aqueous media. Among pteridophytes, most members of the family Pteridaceae and Salviniaceae are more potent as regards removing PTEs [78]. Plants interact with the soil, water, air and natural microbial stimulants involved in removing the contaminants [79]. Plant-based bio-stimulants counteract the adverse effects of pollutants, and improve the antioxidant mechanisms in plant cells that make these plants more resistant to oxidative stresses [80]. Some of the techniques of phytoremediation on the basis of contaminant fate and mechanism of remediation involved are discussed hereunder.

### 5.1. Phytoextraction

**Figure 4:**
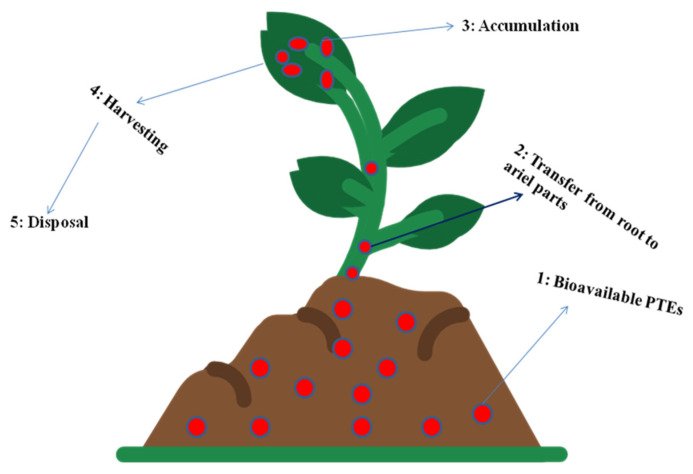
Representation of the major mechanisms underlying phytoextraction.

It exploits the potential of plant roots to exclude, extract and subsequently transport heavy metals to aerial parts [62]. These plants are then removed from the site after being heavily hoarded with PTEs (Figure 4). This technique leaves comparatively less biomass to be disposed of than when excavating the total soil or other media from the site. Phytoextraction can be continuous or induced; in induced phytoextraction, accelerators or chelators are added to the soil to improve metal accumulation over time, whereas in continuous phytoextraction, natural hyperaccumulators that hoard higher levels of the toxic elements over the course of their lifetime are used [81]. Generally speaking, plants that are suitable for phytoextraction develop the following traits: rapid growth rate, high biomass, and potential to withstand higher PTE concentrations [44]. Hyperaccumulators are better suited for remediation because they accumulate 10 to 500 times more PTEs than other common plants [44]. A total of 450 plant species were found to accumulate PTEs, representing about 0.2% of total plant diversity on earth [82], with the majority being members of the plant families Brassicaceae, Lamiaceae, Euphorbiaceae, Asteraceae, and Scrophulariaceae [83,84]. Phytoextraction can be halted by the low solubility of PTEs in the media and lower availability for the plant uptake [85]. However, acidified organic products, such as cow dung, could be effective for phytoextraction of some PTEs, such as Pb and Cd [86].

### 5.2. Rhizofiltration

**Figure 5:**
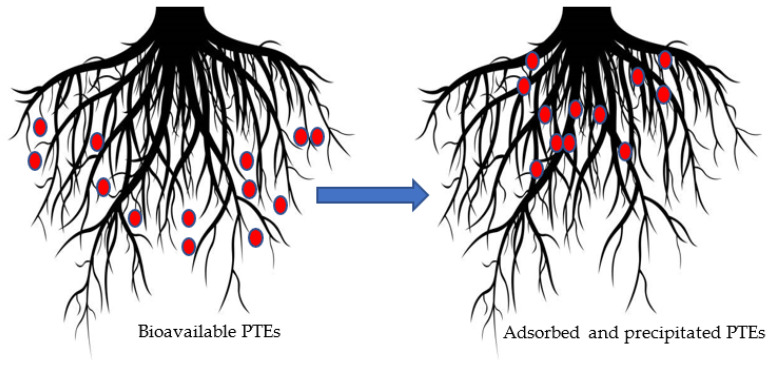
Mechanism underlying rhizofiltration.

In rhizofiltration, contaminants in media surrounding the root zone are adsorbed or precipitated on or into plant roots (Figure 5). Rhizofiltration firstly causes repression of noxious waste, and then the contaminants are accumulated on or within the plant. By physically removing the plants and accumulated contaminants, the concentration of contaminants dwindles from the site. Rhizofiltration confiscates pollutants that contaminate water bodies through industrial discharge and agricultural runoff [87]. Rhizofiltration, a hydroponically based green technology, uses aquatic and terrestrial plants to absorb and precipitate PTEs from wastewater to treat water. Without soil, plants are grown in greenhouses in water, and to help the plants adapt to their surroundings, contaminated water from the sites is used. After being planted, the plants are placed on contaminated ground, where their root systems absorb both the contaminants and water. The plants are harvested along with the roots once the roots have become saturated with the contaminant [88]. PTEs such as lead, cadmium, cobalt, copper, uranium, and arsenic can be effectively removed using this technique but cannot be effectively removed using conventional methods [89].

### 5.3. Phytostabilization

**Figure 6:**
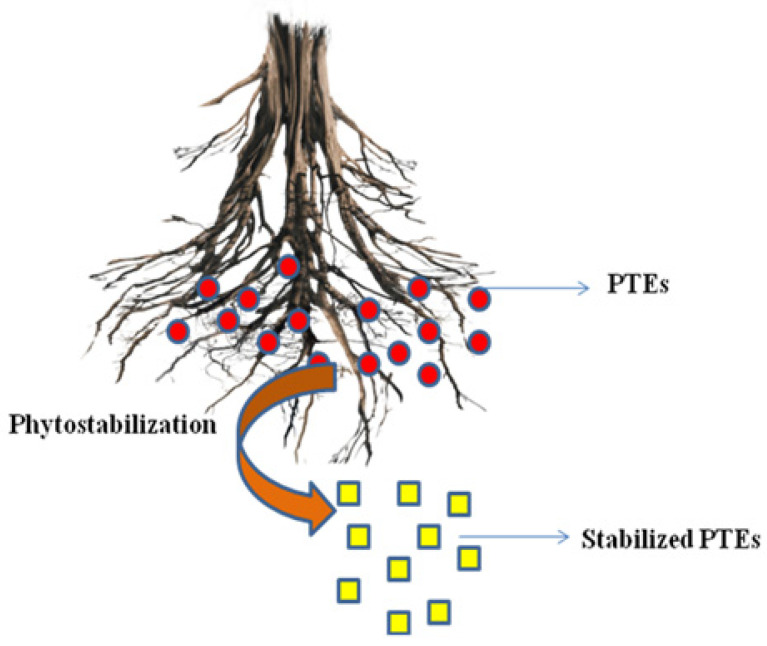
Major mechanisms underlying phytostabilization.

Phytostabilization is also known as phytoimmobilization or phytosequestration. It is defined as halting soil pollutants by preventing relocation of contaminants through wind and water erosion, leaching, and soil dispersion, via absorption and accumulation by root systems of plants [90] (Figure 6). Phytostabilization can alter the solubility and mobility of contaminants or affect the dissociation of organic compounds and, thus, prevent their dispersion through wind or water [81]. Erosion and leaching of contaminants can facilitate air- or waterborne pollution of nearby locations. In phytostabilization, plant root exudates cause pollutant immobilization, thus reducing the availability of soil contaminants [91]. Precipitation, sorption, metal valence reduction, and complexation all result in the phytostabilization of PTEs [58]. The following traits should be present in plants used for phytostabilization: a dense root system, the capacity to withstand soil conditions, ease of establishment and maintenance in field settings, rapid growth, and the capacity for self-reproduction [44]. Several plant species including grasses and willows are effective for phytostabilization owing to their higher tolerance to PTE toxicity [92]. Phytostabilization is generally helpful when PTEs need to be quickly immobilized to avoid contaminating groundwater. Nevertheless, as toxins are still present in the media, ongoing environmental monitoring is necessary, which could be a problem [44].

### 5.4. Phytovolatilization

**Figure 7:**
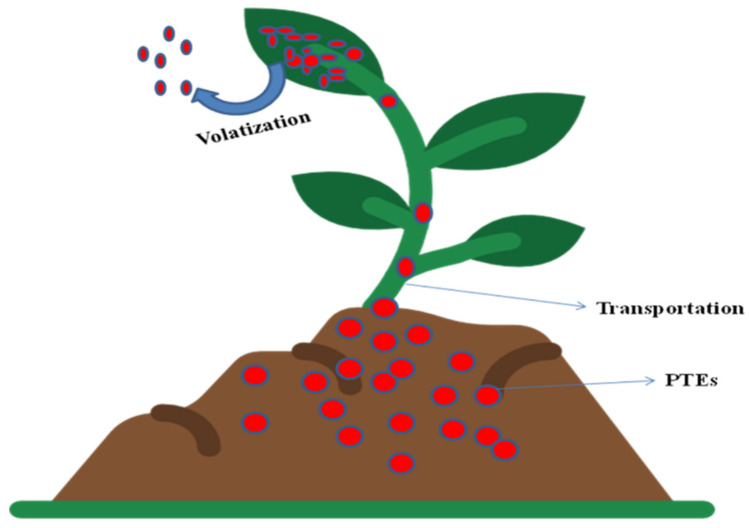
Major mechanisms underlying phytovolatilization.

In phytovolatilization, the soluble toxins are absorbed along with water through plant roots and modified forms of the pollutants are released into the air after metabolism through transpiration [93,94] (Figure 7). This technique is used to purify soils and aquatic media from PTEs such as Se, Hg and As. Phytovolatilization of PTEs in plants involves specific mechanisms governed by specific genes or enzymes. Cystathionine gamma-synthase (CGS) enzymes play a key role in the volatilization of PTEs inside in plants [95]. For the remediation of mercury-contaminated soils, phytovolatilization is preferable as during this process, mercury’s toxic Hg ion is altered into its less noxious elemental form [44]. The drawback of phytovolatilization is that the elemental Hg may be re-deposited in water bodies, which causes anaerobic bacteria to produce methyl-Hg again [44].

### 5.5. Phytorestoration

This process engages the complete remediation of polluted media back to exclusively functioning media. Such type of phytoremediation requires native plant species to return contaminated sites to its natural state. Phytoremediation processes intend to renovate the highly contaminated soils and water bodies back to a validly tolerable level of contamination. An amalgam of phytoremediation techniques can be employed for further effectual environmental restoration. This may aid in concurrently eradicating diverse forms of contaminants or wastes from the exact location [96]. The choice of plant species deployed for remedial purposes is influenced by numerous factors, such as their capability to core with the concerned pollutants and their flexibility to other site-specific factors [97]. The potential to accumulate metals, tolerance to accumulated PTE concentration, rapid growth and high biomass, extensive root systems and palatability to humans and animals are just a few of the traits that plants suitable for phytoremediation should have [98]. There are many factors that affect the uptake and degradation of PTEs by plants, which include the nature of the plant species, rhizosphere, properties of the medium, environmental conditions, bioavailability of contaminants and nature of contaminants. Table 2 presents an inventory of plant species which have been reported to have remediation potential along with their mechanism of actions.

Phytoremediation strategies utilizing such plants surpass traditional technologies and the most significant advantage of phytoremediation over traditional technologies is their lower energy costs and eco-friendly nature [123]. PTEs can change from being highly toxic to being easily volatilized, more water soluble (allowing for leaching removal), less water soluble (allowing for precipitation and easy removal from the environment), or less bioavailable due to a change in their oxidation state [44]. Phytoremediation is environmentally friendly, inexpensive, suitable for soil as well as aqueous media and is easy to execute and maintain. Although owing superiority over conventional methods of remediation, phytoremediation has some limitations.

Application of plant biomass is more effective for low concentrations of pollutants as higher concentrations of contaminants in the media may not allow the plant species to grow/survive. For effective remediation, the contaminants in media must be sufficiently shallow so that plant roots can attain and absorb these contaminants [124]. Ecological exposure issues may arise due to the accumulation of contaminants in plant tissues. The use of alien species for phytoremediation purposes may cause damage to the ecosystem, as alien species may become invasive. Time taken to attain the desired results may be longer compared with other remedial technologies. The nature of biodegradation products is not always known and it may be more toxic sometimes. There are chances of relocation of contamination across media, e.g., from soil to air. Application is limited to certain types of watercourses. There is a possible chance of entry of toxins into the food chains and this may result in biomagnifications.

## 6. Recent Advances in Phytoremediation

Several modern approaches to overcome the abovementioned limitations and to enhance the phytoremediation processes are described below.

### 6.1. Role of Genetic Engineering in Phytoremediation

PTE contamination is a leading environmental problem threatening the survival of plants, animals, and humans, as well as degrading the environment. Phytoremediation is among the utmost economical and eco-friendly techniques to tackle these pollutants that introduce plant biomass into a milieu and let them assimilate/degrade the undesirable particles in their aerial/underground parts. Phytoremediation processes intend to renovate the highly contaminated ecosystems back to a validly tolerable level of contamination. However, hyperaccumulators are small, sluggish, and frequently rare species with insufficient population sizes and extremely constrained distributions [125]. Natural plants have less potential as phytoremediators because of their slow growth, low biomass accumulation, and sensitivity to specific environmental conditions [126]. Additionally, only a small subset of pollutants currently qualify for phytoremediation technologies, necessitating the engineering of phytoremediators with various stacked genes to fulfill the prerequisites of particular sites. Genetic selection can be an important tool through which the remedial efficiency of natural plants can be improved [126]. *Brassica juncea* and *Helianthus annuus* are two examples of naturally existing plant species that have been genetically modified to create transgenic plants for use in phytoremediation processes [127]. Thus, a biotechnological approach holds potential to make rapid and significant changes in growth and development of plants [128]. Application of genetic engineering in phytoremediation assists in developing high biomass content, an intense root system, and highly tolerant plants which can be grown in minimal specified environmental conditions [129]. A fascinating approach that can be used to produce increased biomass is to understand the biosynthetic pathways for phytohormone synthesis and the overproduction of gene-encoding hormones in intriguing plant species. Such plant species would develop rapidly and would encourage greater levels of pollutant absorption for the cleanup of contaminated environments [130]. It has been demonstrated that increased gibberellin production in transgenic plants encourages growth and biomass production over numerous cycles of decontamination [94]. Further, plant genetic engineering can be an effective approach to exploit potential genes involved in metal uptake, translocation, reduction, vacuolar sequestration and volatilization [131]. The proteins involved in metal absorption, translocation, and sequestration are encoded by several plant genes [131,132]. The buildup of metal may grow several times over with the introduction of these genes into potential plants or through thegenetic modification of metal transporters [133]. Heterologous expression of the arsenite antiporter PvACR3, which decreases arsenic buildup and transfer in plant shoots, resulted in the development of transgenic *Pteris vittata* [134]. Further, increased metal tolerance and accumulation in plants may be supported by the effective expression of metallothionein genes [94]. Thus, the use of biotechnology to develop transgenic plants with improved potential for efficient, clean, cheap and sustainable bioremediation technologies is very promising. Transgenic plants show distinctive features due to their genetic makeup and have exceptional potential to contribute to the revitalization process of contaminated environments [135]. Although the potential as well as the number of plant species that can be used for remediation has increased thanks to recent biotechnological advances, there are still some bottlenecks. For instance, genetically modified plants containing the gene for mercuric reductase (*merA*) and the gene for organomercurial lyase (*merB*) are being used for phytovolatiztation, as most natural plants lack this potential. It is possible to insert *merA* and *merB* into plants that detoxify methyl-Hg to elemental Hg. From a regulatory standpoint, it is unacceptable to use plants that have undergone *merA* and *merB* modification. However, the gene foils the entry of methyl-Hg into the food webs, and plants modified with *merB* are more acceptable [44]. Strategies employed should be to stop gene flow into wild species and biosafety concerns must be properly addressed. Further, application of genetic engineering to identify, introduce and express specific genes sequences for resistivity and tolerance in natural phytoremediators into quickly growing plants to control root growth or boost the production of specific plant enzymes is still being thoroughly researched.

### 6.2. Role of Microbes in Phytoremediation of PTEs

Plants and microorganisms have inherent biological capacity that helps them to live under PTE stress and clean the environments polluted with adverse metals. Numerous microorganisms, such as rhizobacteria, mycorrhizae, and yeast, have been reported as important inoculants to increased plant growth performance and phytoremediation process [136].The use of microbial enzymes or genetically engineered microbes that efficiently degrade PTEs is an advancement for the removal of metals from the soil [137,138]. Favorable environmental factors play a key role in the bioremediation of microbes; otherwise, under unfavorable conditions, their role in bioremediations is hampered. It has been studied that increased Zn phytoremediation could be achieved by over expressing SaNramp1, SaIRT1 of *Pseudomonas fluorescens* under metal stress in *Sedum alfredii* [139]. Plant growth-promoting bacteria (PGPB) have been recently used for phytoremidations for removing toxic metals from the soil. PGPB include rhizospheric bacteria, endophytic bacteria and the bacteria that are involved in phytoremediation. The use of PGPB may alter the metal accumulation capacity and restrict its translocation to a different part of the plant through the growth-promoting traits, such as metal resistance, translocation, accumulation, transformation and sequestration, thus reducing the availability of heavy metals in the contaminated soil [140]. Inoculation of *Medicago sativa* with bacterial strains plays an important role in improving seed germination, growth and reduced heavy metal stress by decreasing the antioxidant enzymes and PTEs’ accumulation content, finally improving the phytostabilization process efficiency [136,141]. Mycorrhizae also display their role in phytoremediation by restricting heavy metals on fungal mycelium through a physical barrier, thus reducing their bioavailability, translocation, and bioaccumulation in the plants [136]. Potential of *Klebsiella* sp. strain inoculation in *Vigna radiata* removes Cd, Cu and Pb and induces plant growth under heavy metal stress [142]. The bacteria *Klebsiella* and *Enterobacter* have highest resistance against Cd and Pb tolerance, as revealed by molecular and biochemical mechanisms. These bacteria are the best candidates for bacteria-assisted phytoremediation strategies against As-, Cd-, and Pb-contaminated soils [143]. *Trichoderma asperellum* inoculation in *Suaeda salsa* induced plant growth and reduced the oxidative stress caused by Pb [144]. Metal-binding proteins, such as phytochelatins, metallothioneins, Cd-binding peptides, cysteines and histidines, interact with microbes that help in the phytoremediation of industrial wastewater containing heavy metals. Microbes protect heavy metals stress through compartmentalization, exclusion and the synthesis of binding proteins [145]. Reports showthat microbes such as *Pseudomonas*, *Bacillus*, and *Aspergillus*, when associated with plants such as *Trifolium repens*, *Helianthus annuus*, and *Vallisneria denseserrulata,* have high metal tolerance, and bioremediation potential [146]. Through contemporary biotechnological methods, it is possible to isolate and cultivate such microbes and supplement the soil to improve the phytoremediation using particular plant species.

## 7. Conclusions

The rise in anthropogenic activity, industrialization, and modern agricultural techniques over the past few decades has raised the risk of PTE toxicity to living things. PTE contamination of soil and water has proven disastrous not only for human health but also for plants, aquatic flora and fauna, as well as microbes. One of the most cost-effective and environmentally beneficial ways to deal with these pollutants is through phytoremediation, which involves introducing plant biomass into the environment and allowing the plants to assimilate/degrade the unwanted particles in their aerial and subsurface portions. Phytoremediation is a cost effective, eco-friendly, feasible approach with a wider applicability. However, natural plants have less potential as phytoremediators because of their slow growth, low biomass accumulation, and sensitivity to specific environmental conditions. Thus, for quick and efficient decontamination of PTEs from the environment, it is recommended to combine various existing approaches of phytoremediation techniques coupled with recent biotechnological approaches. However, risk assessment, layman comprehension, acceptance, and awareness of this environmentally friendly technology, as well as networking among scientists, stakeholders, industrialists, governments, and non-governmental organizations, are crucial issues that must be addressed to ensure the successful implementation of a phytoremediation program.

## Figures and Tables

**Table 1:** Sources and effects of the major PTEs on human health.

PTE	Source(s)	Effects	Reference(s)
Arsenic (As)	Pesticides, fungicides, metal smelters	Carcinogen causes skin, lung, liver and bladder cancer. In addition, causes darkening of the skin	[28,29,30]
Barium (Ba)	Rodenticides, pharmaceuticals, cosmetics, diesel engines	Vomiting, abdominal cramps, diarrhea, difficulty in breathing, hypo or hypertension, numbness, paralysis or death	[31]
Cadmium (Cd)	Welding, electroplating, pesticides, fertilizers, batteries, nuclear plants	Stomachache, vomiting, diarrhea, kidney problems, liver damage, fragile bones, cause itai-itai	[24,29]
Chromium (Cr)	Mining, electroplating, textile, tanneries	Carcinogen, nose ulcers, asthma, cough, skin problems, kidney and liver damage	[28,29]
Lead (Pb)	Paint, batteries, pesticides, automobile emissions, mining, burning of coal	Weakness, hypertension, anemia, brain and kidney damage, miscarriage, impotence	[28,29]
Mercury (Hg)	Pesticides, batteries, paper industries	Minimata disease, nervous disorders, kidney damage, tremors, impaired vision and hearing, loss of memory	[28,29]
Selenium (Se)	Mining, agricultural wastes, petrochemicals,	Nausea, vomiting, diarrhea, sclerosis, respiratory tract irritation, bronchitis, stomach pains, bronchial spasms and cough	[32,33]
Silver (Au)	Industries, mining, silver plating,	Blue–grey discoloration of the skin called Argyria, breathing problems, lung and throat irritation, stomach pain, skin problems such as rashes, swelling and inflammation	[34]

**Table 2:** List of the plant species reported for their role in phytoremediation of varied PTEs.

Taxa	Family	Heavy Metal(s)	Media	Mechanism	Reference(s)
*Agrostis capillaris*	Poaceae	As	Wetlands	Phytostabilization	[99]
*A. tenuis*	Poaceae	Pb	Soil	Phytostabilization	[47]
*Amaranthus spinosus*	Amaranthaceae	Cu, Pb and Cd	Soil	Phytostabilization	[91]
*Arabidopsis thaliana*	Brassicaceae	Hg	Soil	Phytovolatilization	[46,100]
*Arthrocnemum macrostachyum*	Amaranthaceae	Cd	Water	Phytostabilization	[29,101]
*Aspalathus linearis*	Fabaceae	Al	Soil	Phytostabilization	[102]
*Astragalus racemosus*	Fabaceae	Se	Soil	Phytovolatilization	[103,104]
*Athyrium wardii*	Athyriaceae	Pb and Cd	Soil	Phytostabilization	[105]
*Atriplex halimus*	Amaranthaceae	Cd	Wetlands	Phytostabilization	[106]
*Atriplex portulacoides*	Amaranthaceae	Zn	Soil	Phytostabilization	[29,35,107]
*Avicennia marina*	Acanthaceae	As	Wetlands	Phytostabilization	[99]
*Azolla filiculoides*	Salviniaceae	Cd, Pb and Cu	Water	Phytoextraction	[108,109]
*A. pinnata*	Salviniaceae	Pb, Hg and Cd	Water	Phytoextraction	[87]
*Brassica juncea*	Brassicaceae	Pb, Se, Zn and Hg	Soil	Phytoextraction	[110]
*B. oleraceae*	Brassicaceae	Cd and Zn	Soil	Phytoextraction	[111]
*B. chinensis*	Brassicaceae	U	Soil	Phytoextraction	[88]
*B. juncea*	Brassicaceae	U	Soil	Phytostabilization	[97,110]
*B. napus*	Brassicaceae	Cd, Cr, Cu, Ni, Pb, Se, and Zn	Soil	Phytovolatilization	[112]
*Callitriche stagnalis*	Plantaginaceae	U	Water	Rhizofiltration	[113]
*Canna glauca*	Cannaceae	As	Water	Phytoextraction	[97]
*Deschampsia cespitosa*	Poaceae	As	Wetlands	Phytostabilization	[99]
*Eleocharis acicularis*	Cyperaceae	Cu, Zn, As, Cd and Pb	Water	Phytoextraction	[99,109]
*Festuca rubra*	Poaceae	Zn, Cd, Hg, Cu	Mine tailings	Phytostabilization	[99,114]
*Helianthus annus*	Asteraceae	U	Soil, Water	Phytostabilization	[97,115]
*Ipomoea aquatic*	Convolvulaceae	Pb and Cr	Water	Rhizofiltration	[115]
*Iris pseuda-corus*	Iridaceae	Cr and Zn	Water	Rhizofiltration	[115]
*Lemna gibba*	Araceae	As	Water	Phytoextraction	[116]
*L. minor*	Araceae	Cd, Se, and Cu	Water, Soil	Phytostabilization	[115]
*Lepironia articulata*	Cyperaceae	Pb	Water	Rhizofiltration	[115]
*Linum usitatissimum*	Linaceae	Cd	Soil	Phytoextraction	[117,118]
*Lolium perenne*	Poaceae	Cu, Pb, Zn, Cr	Soil, Water	Phytostabilization	[97,99,115]
*Ludwigia stolonifera*	Onagraceae	Cd, Zn, Ni, Pb	Water	Phytostabilization	[115]
*Lupinus uncinatus*	Fabaceae	Cd	Soils	Phytostabilization	[119]
*Mentha aquatic*	Lamiaceae	Ni	Water	Rhizofiltration	[115]
*Nelumbo nucifera*	Nelumbonaceae	Cd, Co, Pb, Ni and Zn	Wetlands	Phytoextraction	[115]
*Nicotiana tabacum*	Solanaceae	Cd, Pb, Hg	Soil	Phytostabilization, phytovolatilization	[44]
*Oenanthe javanica*	Apiaceae	Hg	Water	Phytostabilization	[115]
*Phragmites australis*	Poaceae	Ni, Mo, Se, Cu, Pb and Zn	Wetlands	Phytoextraction	[90,97,115]
*Pistia stratiotes*	Araceae	Cd, Cu, Fe, Hg, Mn and Pb	Water	Phytoextraction	[115]
*Plantago major*	Plantaginaceae	Pb	Water	Rhizofiltration	[115]
*Potamogeton natans*	Potamogetonaceae	U, Pb, Cd and Zn	Water	Rhizofiltration	[115]
*P. pectinatus*	Potamogetonaceae	U and Cd	Water	Rhizofiltration	[111,113]
*P. pusillus*	Potamogetonaceae	Cr and Cu	Water	Phytoextraction	[113]
*Pteris vittata*	Pteridaceae	As	Water	Phytoextraction, phytostabilization	[97,115]
*Ricinus communis*	Euphorbiaceae	Cd, Cu, Mn, Pb, and Zn	Soils	Phytostabilization	[120]
*Salix babylonica*	Salicaceae	Cu	Wetlands	Phytoextraction	[121]
*Salvinia biloba*	Salviniaceae	Pb	Water	Phytoextraction	[115]
*Silene vulgaris*	Caryophyllaceae	As	Wetlands	Phytostabilization	[122]
*Triglochin maritime*	Juncaginaceae	Hg	Wetlands	Phytostabilization	[122]
*Typha domingensis*	Typhaceae	Al, Fe, Zn, Hg and Pb	Water	Phytoextraction	[115]
*T. latifolia*	Typhaceae	Mn, Cd, Zn, Co, Ni and Cr	Water	Phytoextraction	[97,115]
*Vallisneria natans*	Hydrocharitaceae	As	Water	Rhizofiltration	[115]

## Data Availability

Not applicable.
